# HSP90 as an emerging barrier to immune checkpoint blockade therapy

**DOI:** 10.18632/oncoscience.554

**Published:** 2022-04-22

**Authors:** Daolin Tang, Rui Kang

**Affiliations:** ^1^Department of Surgery, UT Southwestern Medical Center, Dallas, TX 75390, USA

**Keywords:** HSP90, immune checkpoint, pancreatic cancer, protein degradation

## Abstract

Immunotherapy, especially the use of immune checkpoint inhibitors, has improved overall survival in cancer patients. However, a large proportion of patients initially do not respond to treatment or relapse after a period of response. Heat shock protein 90 (HSP90) is a conserved molecular chaperone that promotes the maturation and folding of substrate proteins involved in many different cellular pathways. Our recent drug screen and functional assay identified HSP90 as a universal control of the protein stability of nuclear transcription factor STAT1 in a variety of different cancer cells, thereby promoting subsequent gene expression of immune checkpoint molecules (IDO1 and PD-L1). *In vivo*, we used different mouse models of pancreatic cancer and demonstrated that targeting HSP90 enhanced the efficacy of PD-1 blockade therapy. These findings establish HSP90 as a targetable vulnerability in immune therapy.

## INTRODUCTION

**Figure 1:**
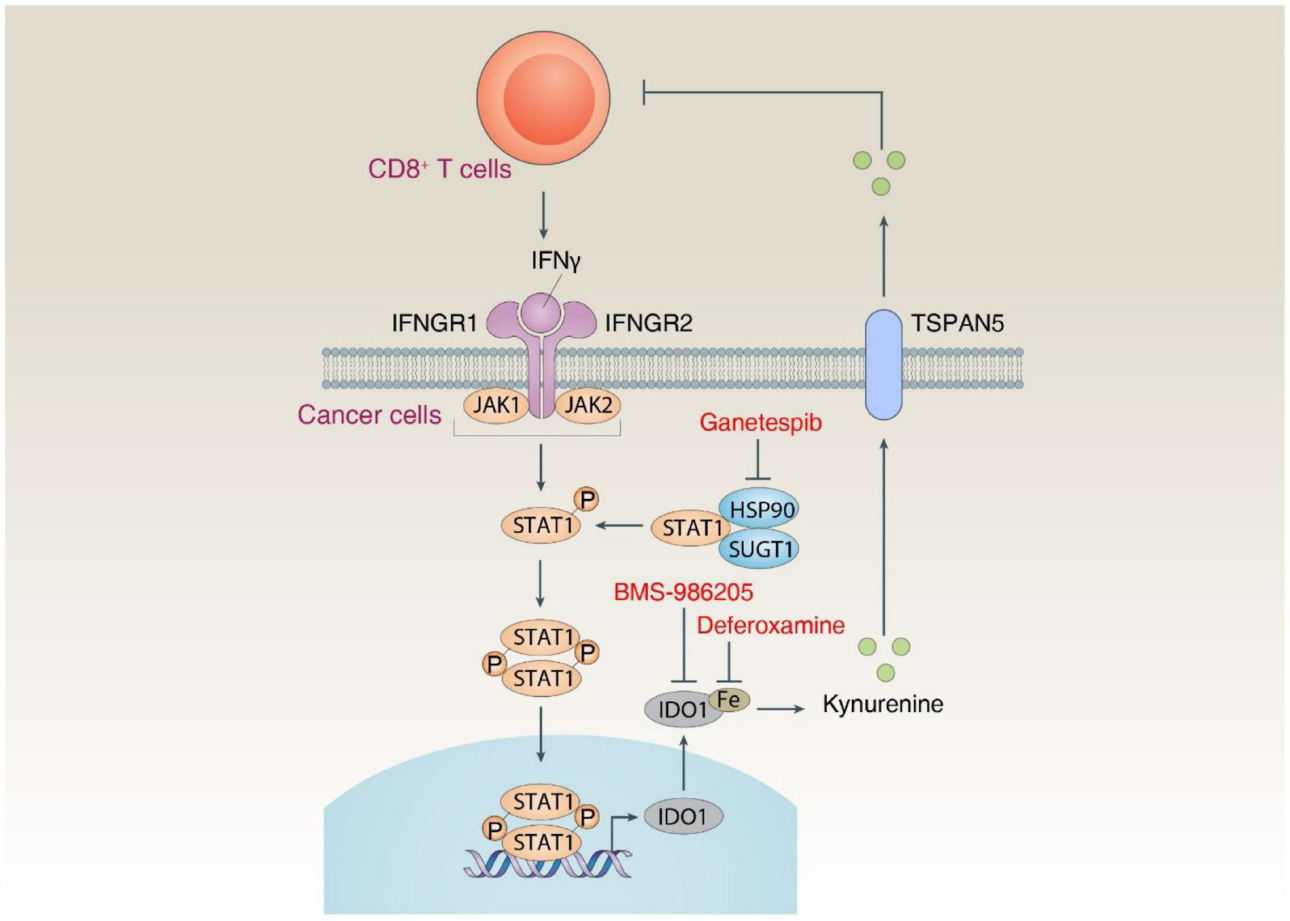
HSP90 mediates IFNγ-induced adaptive immune resistance in pancreatic cancer. Cytotoxic CD8^+^ T cells in the tumor microenvironment can produce IFNγ. IFNγ binds to receptors (IFNGR1 and IFNGR2) on cancer cells, leading to the activation of the JAK-STAT1 pathway and subsequent expression of IDO1. IFNG-induced IDO1 expression requires increased protein stability of STAT1 mediated by the HSP90-SUGT1 chaperone complex. The activity of IDO1 in the production of kynurenine is mediated by iron, while the release of kynurenine is mediated by TSPAN5. Kynurenine is a potent immunosuppressive metabolite of various immune cells, including CD8^+^ T cells.

*KRAS*-driven pancreatic ductal adenocarcinoma (PDAC) is a highly aggressive and lethal malignancy due to lack of early diagnosis and limited response to treatments, including chemotherapy, radiotherapy, and immunotherapy [1]. Immune checkpoint inhibitors (ICIs) are a class of immunotherapy drugs that have improved the treatment of various solid cancers, but not PDAC [2]. Understanding ICI resistance mechanisms may open new directions for improving PDAC treatment strategies [3, 4]. The immune checkpoint molecules programmed cell death protein 1 (PD-1, also known as PDCD1) and programmed death-ligand 1 (PD-L1, also known as CD274) are a receptor-ligand system that interconnects in the tumor microenvironment to block anti-tumor immune responses [2]. We recently revealed that HSP90 is a previously unrecognized target for overcoming adaptive resistance to PD-1 blockade therapy in PDAC (Figure 1) [5].

Immune checkpoints refer to a series of immune regulatory pathways mediated by different molecules, mainly expressed on the cell membrane, but can also exist inside cells. They play different roles in tumor immune surveillance evasion by shaping communication in cancer cells or cells in the tumor microenvironment [2]. In a subcutaneous B6 mouse model transplanted with KPC cells (a mouse PDAC cell line derived from mice with *Kras* and *Tp53* mutations), we first observed that cytosolic indoleamine 2,3-dioxygenase 1 (IDO1), a heme enzyme that catalyzes the oxidation of tryptophan to kynurenine, was selectively up-regulated in tumors from anti-PD-1 (αPD-1) resistance group [5]. Other common immune checkpoint molecules were unchanged compared to the αPD-1-sensitive group [5], suggesting that inducible IDO1 is a biomarker of adaptive resistance in PDAC cells to PD-1 blockade.

To identify new compounds that can block IDO1 protein expression, we performed a small-scale screen of approximately 450 drugs in the human PDAC cell line CFPAC1 after treatment with interferon gamma (IFNG/IFNγ) in the absence or presence of 10 μM targeted compounds. Surprisingly, 71% of the compounds that blocked IFNγ-induced IDO1 expression were heat shock protein 90 (HSP90) inhibitors [5]. Further, six HSP90 inhibitors, including luminespib, ganetespib, SNX-2112, PF-04929113, HSP990 and XL888, showed high activity in the nanomolar range, blocking not only IFNγ-induced IDO1 expression but also IFNγ-induced PD-L1 expression [5]. Our immunoprecipitation in combination with mass spectrometry and mutational analysis demonstrated that HSP90 formed a complex with its partner SGT1 homolog, MIS12 kinetochore complex assembly cochaperone (SUGT1) to prevent degradation of its client signal transducer and activator of transcription 1 (STAT1) [5]. Because STAT1 plays a central role in transcriptional regulation of multiple immune checkpoint molecules [4, 6], our findings established an upstream pathway that promoted the expression of IDO1 and PD-L1 by maintaining the stability of STAT1 protein.

We also highlighted that iron was necessary for the function of IDO1 in mediating the production of kynurenine, a metabolite that triggers apoptosis in immune cells. Iron is a cofactor for many enzymes, including IDO1, which is required for cell growth under physiological conditions [7]. We demonstrated that iron, but not other metal ions, enhanced IDO1-mediated kynurenine production, while iron chelator desferoxamine was able to enhance CD8^+^ T cell activity [5]. The release of kynurenine is mediated by the transmembrane protein tetraspanin 5 (TSPAN5) [5]. These findings support that iron metabolism in the tumor microenvironment can influence antitumor immune responses, although excess iron production induces regulated cell death, such as ferroptosis, to kill tumors [8]. Additional work is required to identify key checkpoints for iron-mediated cell survival and cell death.

Our experiments in animal models of PDAC (transgenic or subcutaneous transplant models) demonstrated that targeting the HSP90-IDO1 pathway could reverse adaptive resistance to αPD-1 treatment [5]. Specifically, we tested two drugs used in clinical trials, ganetespib and BMS-986205, to block HSP90 and IDO1, respectively. They enhanced the anticancer activity of αPD-1 [5]. Similarly, the iron chelator deferoxamine also showed strong immunostimulatory activity in enhancing CD8^+^ T cell responses in αPD-1 treatment [5]. In contrast, targeting the HSP90-IDO1 pathway did not cause significant histotoxicity in liver and kidney [5].

In conclusion, we establishe a novel approach to enhance the activity of ICIs by targeting HSP90-mediated STAT1 protein stabilization [5]. As HSP90 is an evolutionarily conserved molecular chaperone involved in stabilizing and activating multiple proteins [9], the efficacy, safety, and off-target effects of this novel combination therapy using HSP90 inhibitors and ICIs remain to be further clinically evaluated. Nonetheless, targeted degradation of immune checkpoint proteins may offer other possibilities for tumor immunotherapy [10].
